# Sterigmatocystin, 5-Methoxysterigmatocistin, and Their Combinations are Cytotoxic and Genotoxic to A549 and HepG2 Cells and Provoke Phosphorylation of Chk2, but not FANCD2 Checkpoint Proteins

**DOI:** 10.3390/toxins13070464

**Published:** 2021-06-30

**Authors:** Sanja Dabelić, Domagoj Kifer, Daniela Jakšić, Nevenka Kopjar, Maja Šegvić Klarić

**Affiliations:** 1 Department of Biochemistry and Molecular Biology, Faculty of Pharmacy and Biochemistry, University of Zagreb, 10000 Zagreb, Croatia; sanja.dabelic@pharma.unizg.hr; 2 Department of Biophysics, Faculty of Pharmacy and Biochemistry, University of Zagreb, 10000 Zagreb, Croatia; domagoj.kifer@pharma.unizg.hr; 3 Department of Microbiology, Faculty of Pharmacy and Biochemistry, University of Zagreb, 10000 Zagreb, Croatia; djaksic@pharma.unizg.hr; 4 Mutagenesis Unit, Institute for Medical Research and Occupational Health, 10000 Zagreb, Croatia; nkopjar@imi.hr

**Keywords:** Sterigmatocystin, 5-methoxysterigmatocystin, checkpoint proteins, cytotoxicity, genotoxicity, mycotoxin interactions

## Abstract

Sterigmatocystin (STC) and 5-methoxysterigmatocystin (5-M-STC) are structurally related mycotoxins with cytotoxic and genotoxic properties. In the present study, we hypothesized that DNA damage induced by non-cytotoxic concentrations of single and combined mycotoxins could alter the phosphorylation of the checkpoint proteins Chk2 and FANCD2 (ELISA) in HepG2 and A549 cells. The cytotoxic potential (MTT test) of single and combined STC and 5-M-STC, the nature of their interaction (additivity, antagonism, or synergy) and DNA damage level (alkaline comet assay) in HepG2 and A549 cells were also investigated. All experiments were performed after 24 h of mycotoxin treatment. 5-M-STC was 10-folds more cytotoxic than STC to both HepG2 and A549 cells. Both mycotoxins are genotoxic to HepG2 and A549 cells by inducing both double and single DNA strand breaks that activate Chk2 (especially in HepG2 cells) but not the FANCD2 protein. STC exerted higher genotoxic potential than 5-M-STC in HepG2 and A549 cells when both toxins were applied individually at the same concentration. Dual combinations of non-cytotoxic mycotoxin concentrations showed additive to antagonizing cytotoxic and genotoxic effects. The absence and low activation of checkpoint proteins during prolonged exposure to non-cytotoxic concentrations of STC and 5-M-STC could support cell proliferation and carcinogenesis.

## 1. Introduction

Streigmatocystin (STC) and 5-methoxysterigmatocystin (5-MET-STC) are closely related polyketide mycotoxins mainly produced by diverse species of *Aspergillus* section *Nidulantes* series *Versicolores* [1,2,3,4]. STC can also be produced by the aflatoxigenic Aspergilli section *Flavi* but these species do not accumulate STC in large amounts due to its conversion into aflatoxin [5]. Since Aspergilli series *Versicolores* co-occur frequently as primary colonizers in damp dwellings [1,6,7], both STC and 5-MET-STC can be deposited in indoor dust through viable and dead spores and mycelial fragments of producer strains. Several studies reported STC’s presence in the dust of occupational settings and water-damaged indoors but very few studies addressed the presence of its derivate 5-MET-STC [1,4,6,7,8]. The occurrence of STC in foods seems to be rare; it has been detected at relatively low concentrations in cereals, beer, cheese, and spices as well as in traditional Chinese medicinal plants [5,9,10]. So far, there have been no available reports on 5-MET-STC occurrence in foods.

As a precursor of aflatoxin, STC is structurally related to this carcinogenic mycotoxin. Upon ingestion, both are activated by the liver cytochrome P450 system to reactive an epoxide that forms DNA adducts with guanine [5]. Although STC induces tumours, including hepatocellular carcinomas, hemangiosarcomas of the liver, and pulmonary adenomas, it has been classified as a 2B carcinogen (possible human carcinogen) by the International Agency for Research on Cancer [11]. A comparison of the benchmark dose for a response of 10% extra risk (BMD_10_) of STC for the occurrence of haemangiosarcomas and that of aflatoxin for the occurrence of hepatocellular carcinomas suggested that the carcinogenic potency of STC is approximately three orders of magnitude lower than that of aflatoxin [5]. STC exerted cytotoxic and genotoxic properties in several cell lines of human origin including hepatocellular carcinoma HepG2 cells [12], oesophageal epithelial Het-1A cells [13], lung adenocarcinoma A549 cells, and immortalized bronchial epithelial BEAS-2B cells [14]. Data on the toxic properties of 5-M-STC are scarce. While STC is activated by CYP enzymes producing reactive epoxide, no such metabolite was detected with 5-M-STC [15]. In a TA100 *Salmonella typhimurium* mutagenicity assay, 5-M-STC exerted mutagenic properties in the presence of metabolic activation [16]. Cytotoxic and genotoxic effects of 5-M-STC have been reported in A549 cells [17,18]. 

The serine/threonine kinase Chk2 is the transducer kinase involved in spreading the DNA damage signal through the phosphorylation of effector proteins involved in DNA repair, cell cycle regulation, p53 signalling, and apoptosis [19]. Upon single and/or double strand DNA damage, Chk2 is phosphorylated at Thr68 by serine/threonine protein kinase ATM (ataxia telangiectasia mutated), but it may also be activated by DNA-dependent protein kinase (DNA-PKcs) when DNA damage occurs during mitosis [19,20,21]. Fanconi anaemia (FA) group D2 or FANCD2 is a protein of the FA signalling transduction pathway. FA-pathway-deficient cells display spontaneous DNA strand breaks under normal growth conditions and defect of DNA damage checkpoint activation in response to DNA damage or replication stress [22]. Upon activation, FANCD2 is monoubiquitinated and works in cooperation with other FA and non-FA proteins to repair DNA damage [23]. FANCD2 also function as a transducer of ATM signalling; the phosphorylation of FANCD2 at Ser222, initiated by ATM, contributes to arresting cells in the S phase of the cell cycle [24]. The DNA damage repair function of FANCD2 is crucial in cell cycle arrest and resumed proliferation as well as in elimination of over-damaged cells via apoptosis [23,25].

Cell cycle arrest may be one of the underlying mechanisms of STC genotoxicity [9]. In Het-1A cells, STC provoked DNA damage and triggered G2 phase arrest by up-regulating G2/M regulatory proteins Cdc25C, Cdc2, and cyclin B1 [13]. In BEAS-2B and A549 cells, STC induced DNA damage and cell cycle arrest related to altered expression of cyclin-dependent kinase-CDK1 and cyclin B1, which all may contribute to lung carcinogenesis [14]. So far, there have been no available data on 5-M-STC alterations of cell cycle related proteins.

Since STC and 5-M-STC are structurally related mycotoxins with cytotoxic and genotoxic properties in vitro, we hypothesized that DNA damage induced by sub-cytotoxic concentrations of individual and combined mycotoxins might alter Chk2 and FANCD2 phosphorylation and/or expression in HepG2 and A549 cells. The cytotoxic potential of single and combined STC and 5-M-STC, the type of their interaction (additivity, antagonism, or synergy) as well as DNA damage level evoked by their sub-cytotoxic concentrations in HepG2 and A549 cells were also explored.

## 2. Results

Cytotoxicity (MTT test), genotoxicity (alkaline comet test), and expression of Chk2 as well as FANCD2 phosphorylation and expression profiles (cell-based ELISA) were determined in human lung adenocarcinoma A549 cells and hepatocellular carcinoma HepG2 cells upon 24 h exposure to single and combined STC and 5-M-STC.

### 2.1. Cytotoxicity 

**Figure 1:**
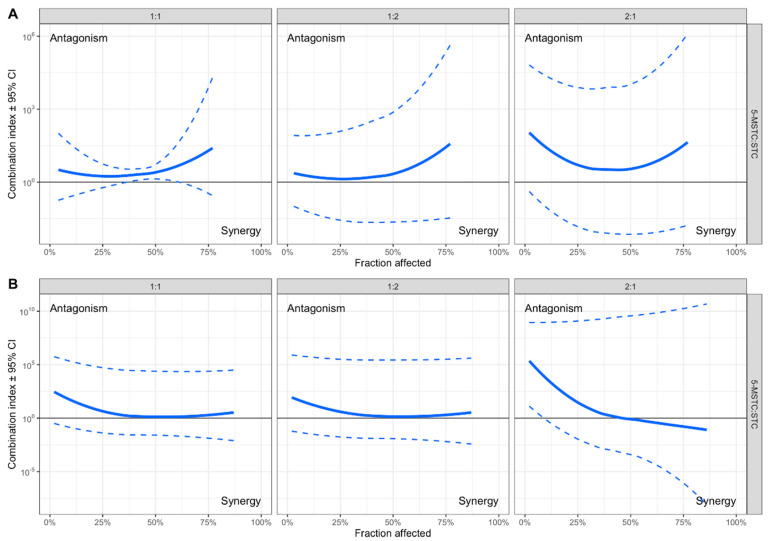
STC and 5-M-STC interactions in HepG2 (**A**) and A549 (**B**) cells are shown as combination indices (CI, thick blue lines) with 95% confidence intervals (dashed blue lines). In HepG2 cells, STC and 5-M-STC showed an additive effect (ratios 1:1, 1:2 and 2:1), while the ratio 1:1 in a narrow range around IC_50_ was antagonistic. In A549 cells, STC and 5-M-STC showed additive (ratios 1:1, 1:2 and 2:1) and antagonistic effects (ratio 2:1 at <IC_10_). Y axis is log_10_ transformed.

According to cell viability upon 24 h treatment with mycotoxin concentrations ranging from 0.1 to 150 µM (Table 1) as well as the IC_50_ (inhibitory concentration for 50% of cells) obtained in the MTT test, HepG2 cells were significantly more sensitive than A549 cells to both mycotoxins with 5-M-STC having about 10 times the cytotoxic potential of STC (Table 2). The means of IC_50_ upon treatment with toxin mixtures in different ratios of IC_50_ obtained by single mycotoxin treatment are presented in Table 3. Results shows that dual-toxin mixtures decreased cell viability to IC_50_ values similar (in HepG2 cells) or lower (in A549 cells) to the IC_50_ obtained after treatment with single 5-M-STC. The nature of the interaction between two mycotoxins is defined with the combination index (CI) according to Chou and Talalay [26] with the addition of the 95% confidence interval as a measure of uncertainty in CI estimation [27]. Two mycotoxins interact additively if the value of 1 is included in the 95% confidence interval of CI; alternatively, if the lower limit of 95% confidence interval is larger than 1 there is antagonistic interaction; and finally, if the higher limit of 95% confidence interval is lower than 1, mycotoxins act synergistically. In HepG2 cells (Figure 1A), STC and 5-M-STC showed mainly an additive effect, and the only exception was antagonism for a 1:1 ratio in a narrow range around IC50. In addition, in A549 cells (Figure 1B), a combination of STC and 5-M-STC exerted dominant additive effects and an antagonism was obtained only for a 2:1 ratio at 10% of inhibited cells. 

### 2.2. Genotoxicity

**Figure 2:**
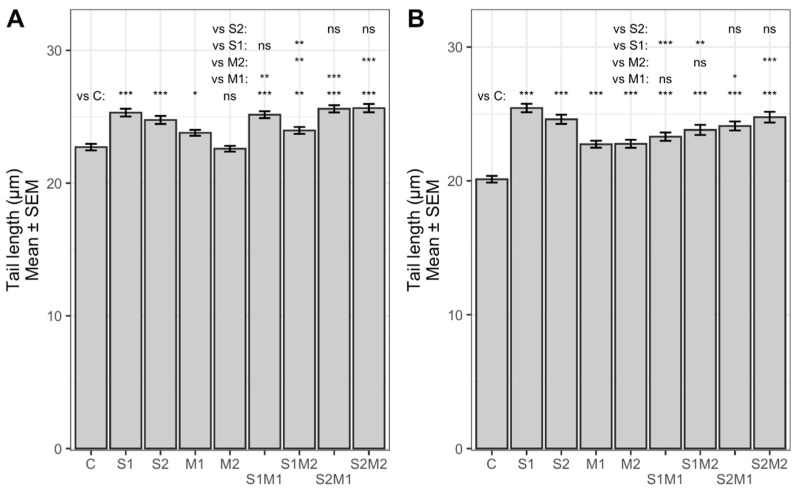
STC and 5-M-STC genotoxic action presented as back-transformed mean of log_10_ TL in HepG2 (**A**) and A549 (**B**) cells. Control cells (C) were treated with 0.09% DMSO (A) and 0.2% DMSO (B). Abbreviations (A): S1 = STC 1 µM; S2 = STC 3 µM; M1 = 5-M-STC 0.1 µM; M2 = 0.3 µM. S1 = STC 3 µM; S2 = STC 12 µM; M1 = 5-M-STC 1 µM; M2 = 3 µM. Abbreviations (B): S1 = STC 3 µM; S2 = STC 12 µM; M1 = 5-M-STC 1 µM; M2 = 3 µM. Statistically significant differences between the groups are emphasized with asterisks coded by *p* values: *** < 0.001 ≤ ** < 0.01 ≤ * < 0.05. ns: not significant.

**Figure 3:**
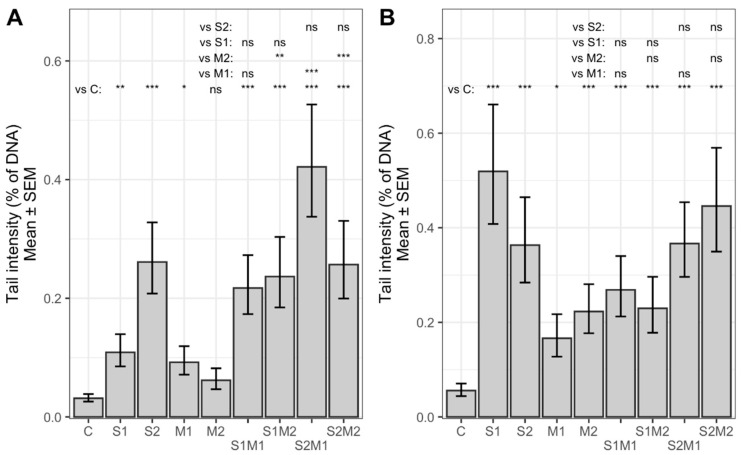
STC and 5-M-STC genotoxic action presented as back-transformed mean of log_10_ TI in HepG2 (**A**) and A549 G2 (**B**) cells. Due to the presence of zeros in TI data, to all TI values were added to the half of the minimum non-zero value. Back transformation accounted for the latter addition. Control cells (C) were treated with 0.09% DMSO (A) and 0.2% DMSO (B). Abbreviations (A): S1 = STC 1 µM; S2 = STC 3 µM; M1 = 5-M-STC 0.1 µM; M2 = 0.3 µM. S1 = STC 3 µM; S2 = STC 12 µM; M1 = 5-M-STC 1 µM; M2 = 3 µM. Abbreviations (B): S1 = STC 3 µM; S2 = STC 12 µM; M1 = 5-M-STC 1 µM; M2 = 3 µM. Statistically significant differences between the groups are emphasized with asterisks coded by *p* values: *** < 0.001 ≤ ** < 0.01 ≤ * < 0.05. ns: not significant.

The alkaline comet assay revealed that single STC (S1, S2) and 5-M-STC (M1, M2) and their combinations (S1M1, S1M2, S2M1, S2M2) applied at subcytotoxic concentrations for 24 h were genotoxic to both A549 and HepG2 cells (Figure 2 and Figure 3). Tail length-TL (Figure 2) was significantly increased in all treatment groups in both cell lines (22–25.8 µm) as compared to control (20 µm in A549 cells, 22 µm in HepG2) (*p* < 0.05, *p* < 0.01, *p* < 0.001). Lower concentration of single STC (S1) in A549 cells evoked a higher increase in TL (25.8 µm) with respect to combinations S1M1 and S1M2 (22 and 23 µm) in A549 cells (*p* < 0.01, *p* < 0.001). Similarly, S1 in HepG2 produced higher TL (25 µm) compared to S1M2 (23 µm) (*p* < 0.01). Both concentrations of 5-M-STC applied alone produced significantly lower TLs (22 and 23 µm) in comparison with combinations S2M1 and S2M2 (around 24 µm) in both cell lines (*p* < 0.05, *p* < 0.001). Tail intensity-TI (Figure 3) was also increased in all treatment groups (0.06 to 0.53%) with respect to control cells (about 0.05%) (*p* < 0.05, *p* < 0.01, *p* < 0.001); the highest TI was obtained in A549 cells treated with lower concentrations of single STC (0.52%). In HepG2 cells, 5-M-STC (M1, M2) alone evoked significantly lower TIs (below 0.1%) compared to combinations S1M2 (0.24%) (*p* < 0.01), S2M1 (0.42%) and S2M2 (0.26%) (*p* < 0.001). It is hard to conclude the nature of interactions of STC and 5-M-STC, but based on TLs and TIs evoked by single STC and 5-M-STC in comparison with their dual combinations, an additive to antagonising genotoxic effect could be expected. 

### 2.3. Determination of Relative Chk2 and FANCD2 Phosphorylation and Protein Expression Level

**Figure 4:**
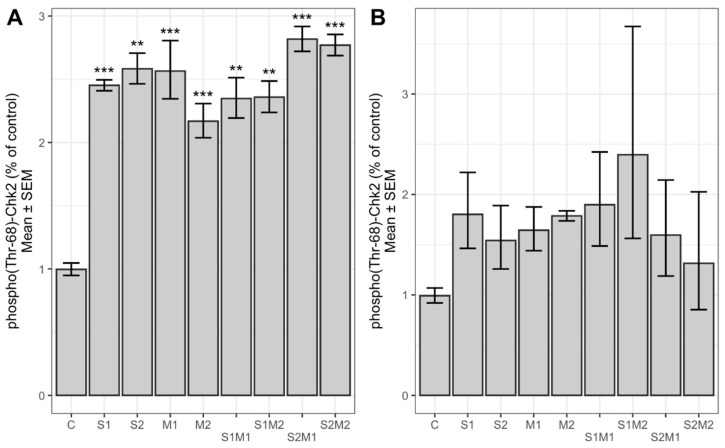
Levels of phosporylated Chk2 (Thr-68) in HepG2 (**A**) and A549 G2 (**B**) cells. Control cells (C) were treated with 0.09% DMSO (A) and 0.2% DMSO (B). Abbreviations (A): S1 = STC 1 µM; S2 = STC 3 µM; M1 = 5-M-STC 0.1 µM; M2 = 0.3 µM. S1 = STC 3 µM; S2 = STC 12 µM; M1 = 5-M-STC 1 µM; M2 = 3 µM. Abbreviations (B): S1 = STC 3 µM; S2 = STC 12 µM; M1 = 5-M-STC 1 µM; M2 = 3 µM. Statistically significant differences between the groups are emphasized with asterisks coded by *p* values: *** < 0.001 ≤ ** < 0.01.

**Figure 5:**
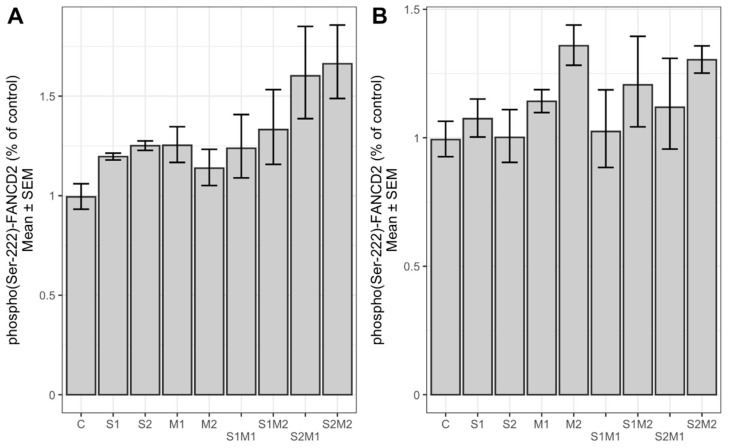
Levels of phosporylated FANCD2 (Ser-222) in HepG2 (**A**) and A549 G2 (**B**) cells. Control cells (C) were treated with 0.09% DMSO (A) and 0.2% DMSO (B). Abbreviations (A): S1 = STC 1 µM; S2 = STC 3 µM; M1 = 5-M-STC 0.1 µM; M2 = 0.3 µM. S1 = STC 3 µM; S2 = STC 12 µM; M1 = 5-M-STC 1 µM; M2 = 3 µM. Abbreviations (B): S1 = STC 3 µM; S2 = STC 12 µM; M1 = 5-M-STC 1 µM; M2 = 3 µM.

We investigated the activation of proteins Chk2 and FANCD2, involved in the regulation of the cell cycle, by the cell-based ELISA method. As shown in Figure 4, the level of phosphorylated (Thr-68) Chk2 was significantly increased in HepG2 cells upon 24 h treatment with single STC and 5-M-STC and their combinations with respect to control (*p* < 0.05, *p* < 0.01, *p* < 0.001). In A549 cells (Figure 4), the expression of phospho(Thr-68)-Chk2 increased but without significant difference with respect to control. In both cell lines, phospho(Ser-222) FANCD2 was not significantly changed compared to controls, although an increasing trend was more pronounced in HepG2 than in A549 cells (Figure 5). The levels of total Chk2 and FANCD2 were not significantly different from controls (data not shown).

## 3. Discussion

Several reports have shown that STC is cytotoxic in a time- and dose-dependent manner in various cell lines including A549, immortalized bronchial epithelial BEAS–2B cells, porcine tracheal epithelial primary PTEC cells, human oesophageal epithelial Het-1A cells, immortalized human gastric epithelial GES-1 cells, and HepG2 cells [12,13,14,15,28,29]. In A549 and BEAS-2B cells, IC_50_ was between 50 and 90 µM, while in HepG2 cells, it was between 3 and 12 µM [12,28], which is in line with the results reported here. On the other hand, the toxic properties of 5-M-STC have been poorly investigated so far; this study as well as our previous report on A549 cells [2,18] are the first to report cytotoxicity of 5-M-STC in vitro. Our results have demonstrated that 5-M-STC is approximately 10-fold more cytotoxic to both A549 and HepG2 cells. This may be explained by the additional methoxy group, an electron-donating group that activates the electrophilic hydroxyl group of 5-M-STC and thus may improve the bioavailability of 5-M-STC. Additionally, the lower cytotoxic potential of STC in comparison to 5-M-STC could be attributed to STC’s unique aggregation properties in water at concentrations between 5 and 10 μM, yielding a strong and specific circular dichroism spectrum [30]. Increasing the concentration of STC also increases the aggregation process, which in turn could decrease cell bioavailability. No such aggregation properties were obtained for structurally related aflatoxin [30] nor for 5-M-STC (unpublished data). Dual combinations of non-cytotoxic concentrations of STC and 5-M-STC showed an additive to antagonizing effect, which could be expected considering their structural similarities and observed aggregation properties. 

Alkaline comet assay showed that both STC and 5-M-STC were genotoxic to HepG2 and A549 cells. Since the alkaline comet assay measures both single and double DNA strand breaks [31], both types of DNA damage could occur upon treatment with STC and 5-M-STC. In terms of TI, the most useful parameter of the comet assay [31,32], STC exerted a higher genotoxic potential than 5-M-STC in both cell lines when both toxins were individually applied at 3 µM. The genotoxic effect was more pronounced in A549 cells. This could also have been due to a lower aggregation rate at lower toxin concentrations [30]. In HepG2 cells, dual mycotoxin combinations provoked a higher TI than single mycotoxins, except for S2M2, which resulted in a similar TI as single S2. In A549 cells, single S1 (3 µM) provoked a much higher TI than its combinations with M1 and M2. These results indicate additive and antagonistic genotoxicity of STC and 5-M-STC, depending on the concentration of the applied STC. The mechanism of DNA damage provoked by STC has not been elucidated yet, while the nature of 5-M-STC genotoxicity is completely unknown. In porcine primary tracheal epithelial cells, STC produced CYP-related reactive metabolites, which might damage DNA, while 5-M-STC was unable to produce such metabolites [15]. Our previously mentioned study showed that STC non-covalently interacts with DNA most probably by intercalation between base pairs, which may induce DNA damage [30]. It is possible that both mechanisms may be in action depending on the bioavailable concentration of STC and CYP activity. The nature of DNA strand breaks provoked by STC and 5-M-STC should be further explored. 

Our results indicate that single and combined STC and 5-M-STC evoked double strand DNA damage in HepG2 and A549 cells that might activate the ATM signalling pathway by increasing the phosphorylation level of Chk2 (Thr-68), a downstream effector of ATM [19]. However, a significant phosphorylation level of Chk2 was obtained only in HepG2 cells, although in A549, an increasing trend of phospho(Thr-68)-Chk2 was observed. Activation of the ATM-Chk2 cell signal is required for the stabilization of p53, which in turn results in the induction of cyclin-dependent kinase (Cdk) inhibitor protein p21 and block cell cycle progression in the G1/S phase [25]. On the other hand, single-strand DNA breaks can activate the ATR kinase, thus inducing a switch from DNA damage signalling by ATM and Chk2 kinases in G1 to additional contributions of ATR and its effector Chk1 kinase in S-phase and G2 [33]. The mechanisms of Chk2-dependent G2/M arrest are like those of G1/S arrest; Chk2 phosphorylates Cdc25C phosphatase resulting in its translocation to the cytoplasm where it cannot activate the cyclinB1/Cdk1 complex, necessary for the G2/M transition [34,35]. In connection to DNA damage and Chk-2 related cell cycle arrest, Chk2 may phosphorylate p53 as well as transcription factor E2F1 to induce both p53-dependent and independent apoptosis [36,37]. Our results support the findings of STC-induced DNA damage and subsequent activation of regulatory proteins, which may lead to cell cycle arrest as seen in A549 and BEAS-2B cells [14]. At relatively low concentrations (6 and 12 µM), a cell cycle checkpoint in the G2/M phase was activated in BEAS-2B cells, while the S phase checkpoint was activated in A549 cells. Opposite to that, at relatively high concentrations (24 µM), both cell lines were blocked in the S and G2/M phase [14]. In human gastric epithelial GES-1 cells, exposure to 3 µM of STC for 24 h induced DNA damage, which subsequently activated ATM-Chk2 and ATM-p53 signalling pathways resulting in G2 arrest [38]. In the same cell line, exposure to 12 µM of STC for 24 h led to Chk1 activation and initial G2 arrest; however, 48 h treatment resulted in Chk1 inactivation, thus promoting checkpoint adaptation and entry of cells into mitosis despite DNA damage [38,39]. Such events may be responsible for STC-induced carcinogenesis upon prolonged exposure to its non-cytotoxic concentrations. To our knowledge, this study is the first to report the activation of Chk2 by STC derivate 5-M-STC. Its possible role in the induction of apoptosis or checkpoint adaptation should be further explored. Combinations of STC and 5-M-STC also increased the expression of phosphorylated Chk2, particularly in HepG2 cells; however, the levels of phospho(Thr-68)-Chk2 upon mycotoxin combinations were similar or lower than after treatments with single mycotoxins, which correspond to DNA damage obtained by comet assay, thus both suggesting STC’s and 5-M-STC’s antagonizing effects.

The absence of FANCD2 activation appears to be an important defect in response to genotoxic events such as DNA crosslinks, single, and double strand breaks [40,41]. FANCD2 can be activated by the phosphorylation of FANCI promoted by the activation of ATR. The phosphorylation of FANCD2 at Ser222 is initiated by ATM, thus contributing to cell cycle arrest in the S phase [23,24]. Furthermore, during tumorigenesis, cancer cells acquired defects in the cell cycle checkpoints by disruption of p53 and E2F1 signalling to increase activity of cyclin-dependent kinase. As FANCD2 is regulated by E2F1, it plays a significant role in the inhibition of cell proliferation [42]. In our study, phospho(Ser-222) FANCD2 was not significantly affected neither in HepG2 nor in A549 cells compared to control; however, a single and dual toxin-dependent increasing trend was evident in HepG2 cells but not in A549 cells. Unchanged levels of phospho(Ser-222) FANCD2 detected in both cell lines after exposure to STC and 5-Met-STC suggest the absence of its activation, which may support cell proliferation upon treatment with non-cytotoxic concentrations of STC and/or 5-M-STC. Finally, this study is the first to report on STC and 5-M-STC effects on phosphorylation of FANCD2 in vitro. 

## 4. Conclusions

5-M-STC is approximately 10-fold more cytotoxic to both HepG2 and A549 cells, whereas HepG2 cells are about 8-fold more sensitive than A549 cells to the cytotoxic effects of both mycotoxins. Differences in the cytotoxic potential of these structurally similar mycotoxins may be related to an additional methoxy group in the 5-M-STC molecule, which may improve the bioavailability of 5-M-STC as well as the aggregation properties of STC, which may reduce the bioavailability of STC. Both mycotoxins are genotoxic to HepG2 and A549 cells by inducing both double and single DNA strand breaks, which activated checkpoint kinase Chk2 (particularly in HepG2 cells) but not the FANCD2 protein. STC exerted a higher genotoxic potential than 5-M-STC in both cell lines when both toxins were applied alone at a concentration of 3 µM, which may be related to STC-CYP-reactive metabolites and/or its DNA intercalation properties. The absence and low-level activation of checkpoint proteins upon prolonged exposure to non-cytotoxic concentrations of STC and 5-M-STC might support cell proliferation and carcinogenesis. Dual combinations of non-cytotoxic concentrations of STC and its derivate 5-M-STC showed an additive to antagonizing cytotoxic and genotoxic effects. The mechanism of their genotoxicity in relation to alterations of cell cycle signalling should be further explored.

## 5. Materials and Methods 

### 5.1. Cell Cultures

Human hepatocellular carcinoma HepG2 cells and human lung adenocarcinoma A549 cells (European Collection of Cell Cultures, Salisbury, UK) were grown in complete cell-culture media, consisting of RPMI 1640, 2 mmol/L glutamine, 10% heat-inactivated FBS, penicillin (100 IU/mL; 1 IU 67.7 μg/mL), and streptomycin (100 μg/mL). Components for cell culture maintenance, including RPMI 1640, fetal bovine serum (FBS), trypsin-EDTA, phosphate-buffered saline (PBS; Ca^2+^ and Mg^2+^ free), penicillin, and streptomycin were from Lonza (Basel, Switzerland). Cultures were maintained in a moisturized atmosphere with 5% CO_2_ at 37 °C and 95% relative humidity. All experiments were performed on cells between passages 3 and 8.

### 5.2. MTT Proliferation Assay

Viability of HepG2 and A549 cells was measured using MTT assay protocol as described in Jakšić Despot et al. [3]. Both cell lines were grown (10^4^ cells/well) in a 96-well flat-bottom microplate in RPMI 1640 medium supplemented with 10% of FBS. Stock solutions of STC and 5-M-STC (Cayman Chemicals, Ann Arbor, MI, USA) were prepared in dimethyl sulfoxide (DMSO) for cell cultures (Sigma-Aldrich, Deisenhofen, Germany). Upon 24 h, cells were treated for the next 24 h with STC or 5-M-STC at concentrations ranging from 0.1 to 150 μM diluted in FBS-free RPMI 1640. Control cells were grown in parallel with or without DMSO (0.2%, 0.7% and 1%) diluted with FBS free RPMI 1640 for 24 h. Following treatment, the medium was removed and 100 μL of MTT tetrazolium salt reagent [3-(4,5-dimethylthiazol-2-yl)-2,5-diphenyltetrazolium bromide] (Sigma-Aldrich, Deisenhofen, Germany) diluted in RPMI 1640 medium without FBS (0.5 mg/mL) was added (V = 100 μL per each well). After 3 h of incubation, the medium was replaced with 150 μL of DMSO (Kemika, Zagreb, Croatia) to dissolve formazan (product of metabolised MTT reagent), and cells were incubated at room temperature on a rotary shaker for 10 min. The absorbance was measured using a microplate reader (Labsystem iEMS, type 1404) at a wavelength of 540 nm. Concentrations that inhibited the viability of 50% of cells (IC_50_) were determined by non-linear regression analysis described in *5.5. Statistical analysis.* Upon determination of IC_50_, cells were treated with dual combinations of STC and 5-M-STC IC_50_ ratios (1:1, 1:2 and 2:1) diluted from 10^0^ to 10^5^. All tests were performed in seven replicates, and results were expressed as percentage of control.

### 5.3. Alkaline Comet Assay

Non-cytotoxic concentrations of STC and 5-M-STC were administered as a single and combined treatment to HepG2 and A549 cells to assess the extent of primary DNA damage. HepG2 cells were treated with single STC (1 and 3 µM) or single 5-M-STC (0.1 and 0.3 µM), or their dual combinations (1 + 0.1 µM, 1 + 0.3 µM, 3 + 0.1 µM, 3 + 0.3 µM); A549 cells were treated with single STC (3 and 12 µM) or single 5-M-STC (1 and 3 µM), or their dual combinations (3 + 1 µM, 3 + 3 µM, 12 + 1 µM, 12 + 3 µM). A standard alkaline comet assay protocol [43] was used with some modifications. Before treatment, the cells were seeded in 6-well plates (10^5^ cells per ml) and incubated (37 °C, 5 % CO_2_, 95 % relative humidity) in a cell medium consisting of RPMI 1640 medium supplemented with 10% of FBS. After 24 h of growth, the cell medium was discarded, and cells were treated with single STC and 5-M-STC and their combinations prepared in FBS free RPMI 1640 as described above. Control treatments were FBS-free RPMI 1640 with and without DMSO (0.09% in HepG and 0.2% in A549 cells). At the end of the incubation period, the cells were washed with cold PBS and trypsinised (trypsin-EDTA). Suspensions of the detached cells were prepared in RPMI 1640 with 10% FBS, centrifuged (200 g, 2 min) and the pellets of medium-free cells were resuspended in fresh RPMI 1640 with 10% FBS. Aliquots (V = 50 μL) of this suspension were mixed with 0.5% LMP agarose, spread onto fully frosted slides, previously pre-coated with 1% and 0.6% NMP agarose, and allowed to solidify on ice for 10 min. Both LMP and NMP agaroses were prepared in Ca- and Mg-free PBS and obtained from Sigma-Aldrich (Deisenhofen, Germany). The microgels were subjected to alkali lysis solution (pH 10) containing NaCl (2.5 mol/L), Na_2_EDTA (100 mmol/L) and Tris (10 mmol/L) supplemented with Triton-X 100 (1%). After the lysis (+4 °C, 60 min), the slides were immersed in denaturation alkaline buffer (NaOH 10 mmol/L, Na_2_EDTA200 mmol/L, pH 13), for 20 min and the same type of buffer was used for electrophoresis (25 V and 300 mA, 20 min). The neutralisation was performed by dripping the slides with by Tris/HCl buffer (0.4 mol/L, pH 7.5). All chemicals were obtained from Sigma-Aldrich (Deisenhofen, Germany), except NaOH and NaCl, which were from Kemika (Zagreb, Croatia). The slides were kept in a humid atmosphere in a dark box at 4 °C until staining with ethidium bromide (20 μg/mL). All experiments were performed in duplicates, and, in each experiment, images of 200 randomly selected nucleoids (100 nucleoids from each of the two replicate slides) were observed by fluorescence microscope (Olympus, Tokyo, Japan) and analysed by Comet assay IV software package (InstemPerceptive Instruments Ltd., Suffolk, Halstead, UK). Only comets with a defined head were scored. Tail length (presented in micrometres) and tail intensity (i.e., percentage of DNA in the comet tail) were selected as measurable indicators of DNA damage [32].

### 5.4. Colorimetric Cell-Based ELISA

Two types of kits based on the same principle were used to determine relative phosphorylation and expression profiles of Chk2 and FANCD2 in HepG2 and A549 cells-CytoGlow™ Chk2 (phosphoThr-68) and CytoGlow™ FANCD2 (phosphoSer-222) colorimetric cell-based ELISA kits (AssayBioTech, Fremont, CA, USA). The analysis was performed according to the product manual. Briefly, 2 × 104 cells/well were seeded in 96-well cell culture plates in complete cell culture media and allowed to adhere for 24 h. The media was discarded and replaced with serum-free media to synchronize cells overnight, prior to treatment. The media was discarded and 200 µL of fresh FBS-free cell culture media supplemented with various concentrations of single STC (S1 = 3 µM and S2 = 12) and 5-M-STC (M1 = 1 µM and M2 = 3) and their dual combinations (S1 + M1 = 3 + 1 µM, S1 + M2 = 3 + 3 µM, S2 + M1 = 12 + 1 µM and S2 + M2 = 12 + 3 µM) was added. Cells were exposed to treatment for the next 24 h. Afterwards, the procedure included all the steps recommended by the manufacturer—fixation in 6% formaldehyde, quenching of endogenous peroxidase activity, blocking, incubation with appropriate primary antibodies (against phosphorylated form or whole protein, regardless on phosphorylation status), incubation with appropriate horse-radish-peroxidase-conjugated secondary antibodies, peroxidase substrate addition, stopping the peroxidase reaction, and cell-washing between steps, when recommended. The absorbance was measured at a wavelength of 490 nm using a microplate reader Victor2-1420 Multilabel Counter (PerkinElmer, Boston, MA, USA). For normalization purposes, also included in the kit, Crystal Violet cell staining that gives absorbance readings proportional to the cell count was applied as the next step. It included washing of the cells, incubation with stain, washing of unbound stain, and solubilization of the stain with SDS-solution, followed by the absorbance reading at 590 nm. All tests were performed in triplicate, and results were expressed as percentage of control (cells exposed to the 0.2% DMSO), normalized to the Crystal Violet stain-determined cell number.

### 5.5. Statistical Analysis

For each batch of cell viability, data obtained by MTT assay (whole experiment replication), mean absorbances of the blanks, were subtracted from all absorbances and the viabilities were calculated by dividing the absorbance observed in wells treated with mycotoxin by the mean absorbance observed in the wells treated only with the same amount of the solvent. The calculated viabilities for each batch, along with the corresponding concentrations of mycotoxins, were used for fitting the four-parameter log-logistic model (R package drc [44]). For each model, the spectre of inhibitory concentrations was estimated, from 1% up to the largest fraction of cells affected. Different batches were summarised using meta-analysis (R package metaphor [45]) where the batch was modelled as a random factor. Combination indices were calculated for the whole spectre of the fraction affected cells according to Chou and Talalay and Anastasiadi et al. accounting for different slopes of dose-response curves [27]. Corresponding 95% confidence intervals (95% CI) were estimated using Monte Carlo simulations (N = 105). If the entire 95% confidence interval was above 1 (in plot 100) or below 1, antagonism or synergy was assumed, respectively. Statistical analysis was performed in R, version 3.6.3. [46].

The observed tail intensity (TI) and tail length (TL) in comet assay were analysed using one-way ANOVA followed by a series of group comparisons between treated groups and control, and combination-treated groups and corresponding single-toxin-treated groups. Prior to analysis, TI and TL were log_10_ transformed to achieve normal distribution. Additionally, due to the presence of zero values within TI, prior to log_10_ transformation, all TI values were increased by the half of minimum non-zero TI value (1.9× 10^−4^% and 1.4× 10^−4^% for HepG2 and A549, respectively). All *p*-values resulting from a series of *post-hoc* t-tests were adjusted according to Bonferroni’s method. Expression levels of phospho(Thr-68)-Chk2 and phospho(Ser-222) FANCD2 resulting from the ELISA experiment were analyzed with one-way ANOVA followed by *post-hoc* Tukey test. All *p*-values less than 0.05 were considered statistically significant. Statistical analysis was performed in R, version 3.6.3, [46].

## Figures and Tables

**Table 1:** Cytotoxic effects of STC and 5-M-STC in HepG2 and A549 cells upon 24 h treatment.

Cell Line	Concentrations (µM)	Cell Viability (%) ± SD	
		STC	5-M-STC
**HepG2**	0.1 0.3 1 3 10 30 60 100 150	100 ± 1.9 106 ± 1.7 90 ± 1.9 64 ± 1.2 45 ± 0.8 25 ± 0.4 24 ± 0.3 18 ± 0.6 26 ± 1.0	80 ± 2.1 60 ± 1.8 45 ± 0.8 39 ± 0.8 31 ± 0.5 27 ± 0.6 25 ± 0.2 20 ± 0.4 20 ± 0.6
**A549**	0.1 0.3 1 3 10 12 30 60 100 150	88 ± 0.6 85 ± 0.5 79 ± 1.0 84 ± 0.4 61 ± 0.7 61 ± 0.8 51 ± 0.7 46 ± 0.4 40 ± 1.0 45 ± 0.3	96 ± 1.0 88 ± 0.7 94 ± 0.6 77 ± 1.0 40 ± 0.3 n.t. 38 ± 0.5 32 ± 0.7 31 ± 1.3 21 ± 0.3

n.t.—not tested.

**Table 2:** Cytotoxic potential of STC and 5-M-STC expressed as IC_50_ in HepG2 and A549 cells.

Cell Line	IC_50_ (STC) μM ± SEM	IC_50_ (5-M-STC) μM ± SEM
HepG2	7.0 ± 1.3	0.7 ± 1.3
A549 *	60.0 ± 1.8	5.5 ± 1.2

* Results of IC_50_ for STC and 5-M-STC were recently reported in our review. Paper by Kifer et al. [18].

**Table 3:** Cytotoxicity of STC and 5-M-STC mixtures in HepG2 and A549 cells.

Cell Line	Mixture Ratio 5-M-STC:STC	IC_50_ Mean ± SEM
HepG2	1:1 1:2 2:1	0.7 ± 1.4 0.6 ± 27.8 0.4 ± 69.4
A549 *	1:1 1:2 2:1	2.1 ± 246.7 1.7 ± 726.6 * 5.3 ± 247545 *

* The standard error of the estimated mean is large because the minimum achieved mean cell viability was above 50%.

## Data Availability

Not applicable.
